# Editorial for the Special Issue on Printable and Flexible Electronics for Sensors

**DOI:** 10.3390/mi11070683

**Published:** 2020-07-15

**Authors:** Jin-Woo Choi, Edward Song

**Affiliations:** 1 School of Electrical Engineering and Computer Science, Louisiana State University, Baton Rouge, LA 70803, USA; 2 Department of Electrical and Computer Engineering, University of New Hampshire, Durham, NH 03824, USA

Printable and flexible electronic materials have gained a tremendous amount of interest both in academia and in industry, due to their potential impact in many areas, including advanced manufacturing, healthcare, diagnostics, wearables, renewable energy, and defense, to name a few. As we were preparing this Special Issue, we had the following questions in mind: What is the future of printable and flexible electronics? How will it advance the sensor technology? What is the current state-of-the-art for wearable sensors? In this Special Issue, we focus on the latest advancements, current challenges, and new opportunities in the world of printable and flexible electronics. Both fundamentals and applications are covered in this issue. The fundamentals include novel materials, manufacturing techniques, and characterization, among others. In terms of applications, chemical and biological sensing, bioelectric signal measurements, strain and pressure sensing, and energy storage were discussed. The goal of this Special Issue is to stimulate the community by addressing the key issues on the topic in the hope that printable and flexible electronics will make a greater impact in our society.

Out of the 12 articles published in this volume of the Special Issue, 11 are original research papers and one is a review article. Six papers were submitted from China, two were from Japan, and one paper each was contributed from Korea, United Kingdom, Portugal, and the United States.

Novel fabrication techniques have been reported in this issue for the development of flexible electronics. For example, a semi-dry electrode containing a flexible body, a foam layer, and a reservoir is developed in order to ensure secure contact between the electrode and the scalp area for electroencephalogram (EEG) measurements. Another example is the use of plasma-induced microjet bubbles for the direct writing of metal patterns on flexible substrates. The advantages here are that this technique does not require the pre-treatment of the surface prior to writing, and a wide variety of metals can be used for writing. A novel electrohydrodynamic jet printing of graphene oxide is also reported, which enabled the layer-by-layer self-assembly of graphene oxide flakes. The nanoflakes were successfully aligned due to the hydrodynamic force, in combination with an externally applied electric field, which further induced the alignment of the flakes through polarization.

In addition to the many fabrication techniques reported here, this issue also covers a broad range of applications for printable and flexible electric devices. A flexible strain sensor made by nanoimprint lithography (NIL) is reported for measuring human body motion by attaching the strain sensor to the skin. The device was sensitive enough to detect joint motion as well as facial muscle movements. In another article, a reduced carboxyl-graphene oxide (rGO-COOH) is used to develop a field-effect transistor for the aptamer-based detection of lead ion. This material is a promising candidate for a self-activated channel material due to its merits of being independent of linking reagents, free from polymer residue, and compatible with printable electronics technology. A flexible blood pulse sensor was also reported. In this article, a novel screen-offset printing method was used, where two types of electronics, the face-up and face-down chips, could be simultaneously mounted on a flexible film substrate. Other examples of applications for the use of flexible electronics in this issue include UV sensors, waveguides, capacitive pressure sensors, and supercapacitors.

Finally, the review article in this issue highlights the recent developments in the topic of lab-on-a-chip (LOC) systems for aptamer-based sensing. As the field continues to advance, the number of published articles on wearable, printable, and flexible LOC devices is also increasing. This article reviews the latest research at the intersection of LOC technology and aptamer-based sensors and its perspective moving into the future. 

We hope that this Special Issue on Printable and Flexible Electronics for Sensors will offer readers a good overview of the current state of the art in this fast-growing area of research as well as an introduction to some of the newest techniques developed in the field.

